# The Treatment of Burkitt Lymphoma With the Berlin-Frankfurt-Münster Protocol With Rituximab and Consolidative Autologous Transplantation

**DOI:** 10.1093/oncolo/oyae017

**Published:** 2024-02-10

**Authors:** Alessandro Broccoli, Lisa Argnani, Gabriele Gugliotta, Cinzia Pellegrini, Beatrice Casadei, Gianmarco Bagnato, Marianna Gentilini, Vittorio Stefoni, Pier Luigi Zinzani

**Affiliations:** IRCCS Azienda Ospedaliero-Universitaria di Bologna, Istituto di Ematologia “Seràgnoli,” Bologna, Italy; Dipartimento di Scienze Mediche e Chirurgiche, Università di Bologna, Bologna, Italy; Dipartimento di Scienze Mediche e Chirurgiche, Università di Bologna, Bologna, Italy; IRCCS Azienda Ospedaliero-Universitaria di Bologna, Istituto di Ematologia “Seràgnoli,” Bologna, Italy; IRCCS Azienda Ospedaliero-Universitaria di Bologna, Istituto di Ematologia “Seràgnoli,” Bologna, Italy; IRCCS Azienda Ospedaliero-Universitaria di Bologna, Istituto di Ematologia “Seràgnoli,” Bologna, Italy; IRCCS Azienda Ospedaliero-Universitaria di Bologna, Istituto di Ematologia “Seràgnoli,” Bologna, Italy; Dipartimento di Scienze Mediche e Chirurgiche, Università di Bologna, Bologna, Italy; IRCCS Azienda Ospedaliero-Universitaria di Bologna, Istituto di Ematologia “Seràgnoli,” Bologna, Italy; Dipartimento di Scienze Mediche e Chirurgiche, Università di Bologna, Bologna, Italy; IRCCS Azienda Ospedaliero-Universitaria di Bologna, Istituto di Ematologia “Seràgnoli,” Bologna, Italy; Dipartimento di Scienze Mediche e Chirurgiche, Università di Bologna, Bologna, Italy; IRCCS Azienda Ospedaliero-Universitaria di Bologna, Istituto di Ematologia “Seràgnoli,” Bologna, Italy; Dipartimento di Scienze Mediche e Chirurgiche, Università di Bologna, Bologna, Italy

**Keywords:** autologous transplantation, BFM regimen, Burkitt lymphoma, methotrexate, rituximab

## Abstract

**Introduction:**

Intensive treatment approaches are required for adult patients with Burkitt lymphoma (BL), although an univocal standard of care still does not exist. The use of frontline autologous stem cells transplantation (ASCT) is debated.

**Patients and Methods:**

Between 2004 and 2020, 50 patients with BL were treated with the Berlin-Frankfurt-Münster (BFM). Treatment plan consisted of 3 blocks, A (ifosfamide, vincristine, methotrexate, etoposide, and cytarabine), B (vincristine, cyclophosphamide, methotrexate, and doxorubicin), and C (vindesine, methotrexate, etoposide, and cytarabine), each repeated twice, every 28 days. Rituximab was given at day 1 each block. Intrathecal prophylaxis was given once per each block. ASCT was scheduled at the end of the 6 blocks after conditioning.

**Results:**

Median age at onset was 38 years (range 16-72); stages III-IV disease was observed in 82% of cases; bulky disease occurred in 44% of the patients, with B-symptoms in 38%. Stem cell harvest was performed in 72% of patients, who all received a subsequent ASCT. The full 6 blocks treatment was completed in 70% of the patients. The overall response rate was 74%, with a complete response rate of 60%. Ten-year overall survival and progression-free survival were 83.7% and 76.0%, respectively, without reaching the median. Ten-year disease-free survival was 80.3%. Grades 3-4 neutropenia, thrombocytopenia, anemia, and mucositis were seen in 96%, 60%, 32%, and 24% of patients. Infections occurred in 60% of patients.

**Conclusion:**

Intensive treatment according to BFM protocol, with rituximab and ASCT, appears feasible, safe, and highly effective in adult patients with BL, as confirmed by long-term survival rates reflecting response maintenance.

## Teaser

This article presents a single-center experience with the Berlin-Frankfurt-Münster protocol, with the addition of rituximab, followed by autologous stem cell transplantation, when feasible, in adult patients with Burkitt lymphoma.

**Implications for Practice**

An age-adapted intensive immunochemotherapy, consisting of a reduced-intensity regimen in elderly patients, with the inclusion of autologous transplantation in younger ones, is effective and feasible in Burkitt lymphoma. A high curability rate is preserved despite the high proportion of advanced stages, which still maintains a major detrimental impact on prognosis. No differences in 10-year outcomes were observed as far as age is concerned, and no increase in toxic consequences was documented in any age categories. Despite autologous transplantation was widely applied, no definite conclusions can be drawn on its consolidative potential, with transplanted and non-transplanted patients displaying superimposable outcomes.

## Introduction

Burkitt lymphoma (BL) is regarded as the most aggressive human neoplasm and, as a consequence of the highly rapid turnover of the neoplastic cells, it often represents a clinical emergency.^1,2^ Besides the endemic variant, firstly described by Denis Burkitt in 1958 and mainly involving children in Equatorial Africa and Papua New Guinea, a sporadic variant and an immunodeficiency-associated variant represent the vast majority of cases described in Western countries.^3^ Both these 2 latter variants have a peak incidence around 40-45 years and around 75 years for the sporadic variant and exhibit a marked tendency to extranodal dissemination.^2,4^

Rapid treatment initiation with adequate support to prevent sepsis, bowel perforation or obstruction (sometimes requiring an emergency surgical approach) and tumor lysis syndrome are the mainstay of patient management.^1^ High dose-intensity regimens applying different classes of chemotherapy agents have been codified for the risk-adapted treatment of children and adolescents affected by BL.^5-7^ The same protocols have been transferred to the frontline treatment of adult patients, however, with an increased toxicity profile that mostly worsens with age and thus frequently yields to dose reductions or delays.^8,9^ Successful results have been obtained by the German multicenter study group for the treatment of adult acute lymphoblastic leukemia (GMALL) with the elaboration of the Berlin-Frankfurt-Münster (BFM) protocol, which has been translated into the treatment of adult patients.^10,11^ Importantly, rituximab was incorporated in the protocol, as its efficacy in patients with BL was confirmed in several independent experiences.^12-14^

Autologous stem cell transplantation (ASCT) has also been evaluated as a consolidative strategy in first remission, mainly following conventional or shorter chemotherapy inductions rather than high dose-intensity protocols.^15,16^ Although results were encouraging, the current application of ASCT as frontline consolidation remains debated, also after BFM.

This article presents a single-center experience with the BFM protocol, with the addition of rituximab, followed by autologous transplantation, when feasible, in adult patients with BL.

## Patients and Methods

### Study Overall Conduct

This is a single-center retrospective study evaluating the safety and effectiveness of autologous transplantation as first consolidation in adult patients affected by BL receiving intensive chemotherapy according to the BFM protocol on an inpatient basis. The schedule contemplates the addition of rituximab at each cycle. It was conducted in accordance with the Declaration of Helsinki and was approved by our institutional board (Ethical Committee AVEC of Bologna, approval id 1043/2021/Oss/AOUBo). Written informed consent was obtained by patients before any study procedure.

All patients aged 18 years or above with a treatment-naïve, histologically proven diagnosis of BL were evaluable. Disease staging was based on total body computed tomography scan and fluorodeoxyglucose (FDG) positron emission tomography scan (PET). Measurable nodal involvement was defined as the presence of lymph nodes with a long axis of at least 1.5 cm and concomitant FDG avidity. Baseline bone marrow (BM) trephine biopsy was always desirable, although considered mandatory in case of peripheral blood lymphocytosis, positive flow cytometry or unexplained cytopenias. It was deemed unnecessary in case of overt BM PET positivity. Besides that, a negative BM histology was required before peripheral blood stem cell mobilization. Patients with an exquisitely leukemic presentation, defined as the presence of peripheral blood and BM disease involvement without measurable lymph nodes, were considered ineligible.

Treatment plan consisted of 3 blocks, block A (with ifosfamide, vincristine, methotrexate, etoposide, and low-dose cytarabine), block B (containing vincristine, cyclophosphamide, methotrexate, and doxorubicin), and block C (with vindesine, methotrexate, etoposide, and high-dose cytarabine). Doses were applied as indicated in Supplementary Table S1. Each block was repeated twice, every 28 days. Patients elder than 60 years did not receive block C, thus blocks A and B were repeated 3 times. Moreover, elderly patients received a reduced dose of ifosfamide (50% of the full dose given in younger patients), methotrexate (33% of the full dose), etoposide (reduced by 40%), cytarabine (reduced by 60%), and vincristine (1 mg, flat dose; Supplementary Table S1). Rituximab was given at day 1 each block at the standard dose of 375 mg/m^2^, regardless of age. Intrathecal prophylaxis (ITP) was given once per each block. Peripheral blood stem cell (PBSC) harvest was planned at recovery after the fourth or fifth cycle; plerixafor could be used if necessary. Autologous PBSC were reinfused at the end of the 6 blocks after BEAM (carmustine, etoposide, cytarabine, and melphalan) conditioning regimen, when feasible.

### Study Endpoints and Statistical Analysis

Primary endpoints were represented by overall response rate and complete response (CR) rate to induction and consolidation. Secondary endpoints were survivals (namely, disease-free survival [DFS], progression-free survival [PFS], and overall survival [OS]) and the incidence and severity of any adverse event (AE) occurring during and right after therapy in all patients receiving at least one cycle of chemotherapy. DFS was determined on all patients in CR as the period between the first documentation of a CR (at any time during the treatment pathway) and the time of clinical disease relapse or death. Patients in remission were censored at the latest available follow-up; relapsing or progressing patients were censored at the time of treatment failure.

AE severity was established as recommended by the National Cancer Institute Common Terminology Criteria for AEs, version 4.03.

Demographics and patients’ characteristics were summarized by descriptive statistics and survival functions were estimated by using the Kaplan-Meier method and compared using log‐rank test or Gehan-Breslow-Wilcoxon test, as applicable. Statistical analyses were performed with Stata 17 (StataCorp LP, TX) and *P*‐values were set at 0.05.

## Results

### Patients

Between 2004 and 2020, 50 consecutive patients with BL were scheduled for BFM, delivered according to their age, and ASCT when clinically feasible. Median age at onset was 38 years (range 16-73); 40 patients (80%) were younger than 60 years, whereas the remaining 10 (20%) were elder than 60. Thirty-eight patients (76%) were male and 12 (24%) female. Four patients tested positive for human immunodeficiency virus (HIV) infection.

Advanced Ann Arbor stages III-IV disease was observed in 82% of cases. Among the 9 patients (18%) with early-stage disease, 5 displayed stage I and 4 stage II. Bulky disease occurred in 44% of the patients (Supplementary Fig. S1) and B-symptoms were reported in 38%. Seventeen patients (34%) had BM disease infiltration. Table 1 summarizes the baseline clinical characteristics according to age.

**Table 1.** Baseline clinical characteristics.

	Age < 60	Age ≥ 60
Patients, *N*	40	10
Male/female, *N*	33/7	5/5
Median age (range), years	33 (16-59)	65 (62-73)
HIV-positive, *N* (%)	3 (7.5%)	1 (10.0%)
Advanced stage (III-IV), *N* (%)	32 (80.0%)	9 (90.0%)
Stage IV, *N* (%)	30 (75.0%)	8 (80.0%)
Bone marrow infiltration, *N* (%)	12 (30.0%)	5 (50.0%)
B-symptoms, *N* (%)	16 (40.0%)	3 (30.0%)
Bulky disease, *N* (%)	15 (37.5%)	7 (70.0%)
Extranodal disease, *N* (%)	33 (82.5%)	8 (80.0%)
Extranodal sites ≥ 2, *N* (%)	14 (35.0%)	4 (40.0%)
Surgery before chemotherapy, *N* (%)	10 (25.0%)	1 (10.0%)

Forty-one patients (82%) presented with extranodal disease (Table 2): the most frequently involved sites were the gastrointestinal tract (stomach and intestine), the pancreas and the skeleton. The spleen was involved in 3 cases, and 3 patients had central nervous system (CNS) dissemination, including meningeal disease. Initial surgical resection of the disease was performed in 11 cases (22%), mainly due to acute abdominal pain (5 cases) or intestinal sub-occlusion (2 cases).

**Table 2.** Affected organs and systems in 38 patients with extranodal dissemination. A single patient may have more than one affected organ. (*) Percentages indicate the prevalence according to system/topography. (**) Numbers indicate how many times each organ was involved.

Gastrointestinal tract	57% (*)
Intestine (small intestine, colon, rectum)	17 (**)
Stomach	11
Pancreas	6
Liver	4
Peritoneum	4
Bone and soft tissues	16%
Skeleton	9
Muscles and soft tissues	3
Head and neck	7%
Tonsil and pharynx	3
Oral cavity	1
Thyroid	1
Uro-genital	7%
Prostate	1
Kidneys	1
Bladder	1
Uterus	1
Ovary	1
Thorax	5%
Lung	3
Pleura	1
Central nervous system, including meninges	4%
Spleen	4%

Cytogenetic evaluation with fluorescence in situ hybridization was performed in 40 patients and conclusive results were available in 33 cases. The t(8;14) was the most frequent cytogenetic marker, being found in 23 out of 33 evaluable cases (70%); t(8;22) was detected in 3 cases (9%) while rearrangements of 8q24 without a specified partner chromosome were reported in 7 cases (21%).

### Chemotherapy Intensity and Compliance

The full 6 blocks treatment was completed in 70% of the patients; 8% received 5 cycles, 6% had 4 cycles and 16% received 3 cycles or less. One patient who received 4 cycles was scheduled to receive a shorter treatment course due to non-bulky stage I disease. Early treatment interruption occurred in 28% of the patients because of disease progression (12%), toxicity (8%), death (4%), or other causes (4%). Among the 4 patients who discontinued because of unacceptable toxicity, 50% were elder than 60 years. Two patients did not receive rituximab because of adverse reaction and early death. ITP was given in 96% of patients.

### Patient Disposition at ASCT

Stem cell harvest was performed in 36 (72%) of patients, who all received a subsequent ASCT. The mean amount of CD34^+^ cells collected was 7.19 × 10^6^/kg. Among patients younger than 60 years, 32 (80%) underwent ASCT. Twenty-three patients were transplanted in CR, 8 in partial response (PR) and 1 under disease progression. Four patients did not receive ASCT because of rapid disease progression or early death; 3 patients were not transplanted due to medical decision (2 due to treatment toxicity and one with stage I disease after the achievement of a CR after 4 cycles, as mentioned above); 1 patient moved to another hospital before ASCT. Four patients elder than 60 (40%) underwent ASCT given their adequate performance status and fitness. All but one of them were transplanted in CR, while 1 underwent ASCT in PR (Supplementary Fig. S2).

### Response to Treatment and Survival Analysis

**Figure 1.**
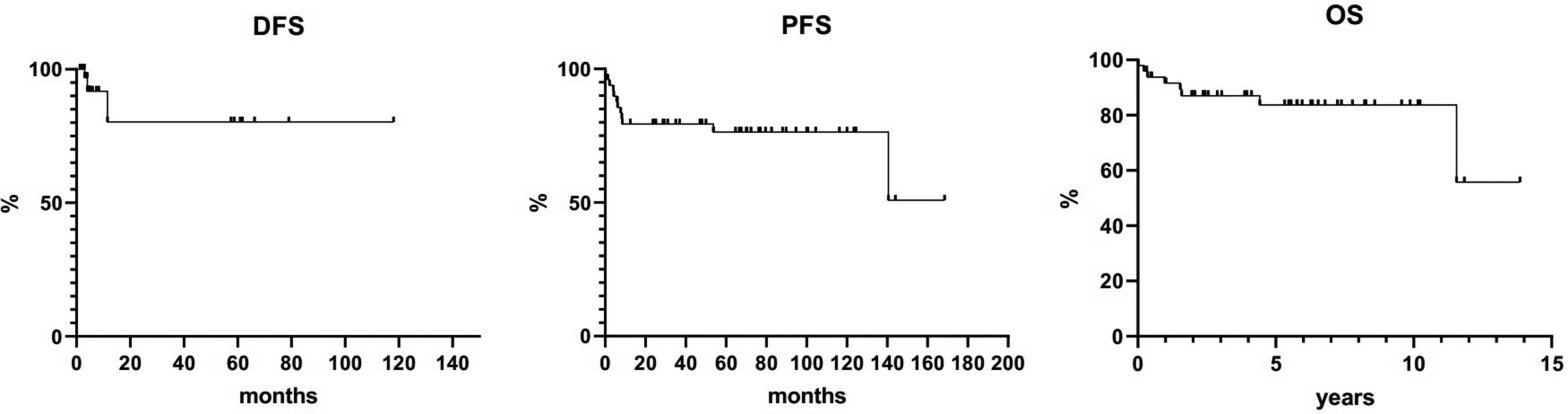
Disease-free survival, progression-free survival, and overall survival curves plotted for the entire population.

The overall response rate was 74%, with a CR rate of 60%. Three patients could not be evaluated because of early progression or death. Ten-year and 14-year OS were 83.7% and 55.8%, respectively, while PFS probability at the same time points was 76.0% and 50.9%, respectively. Ten-year DFS was 80.3% (Fig. 1). Eight patients had died because of disease progression (3 patients), infection or sepsis (4 patients), or cardiac arrest (1 patient).

**Figure 2.**
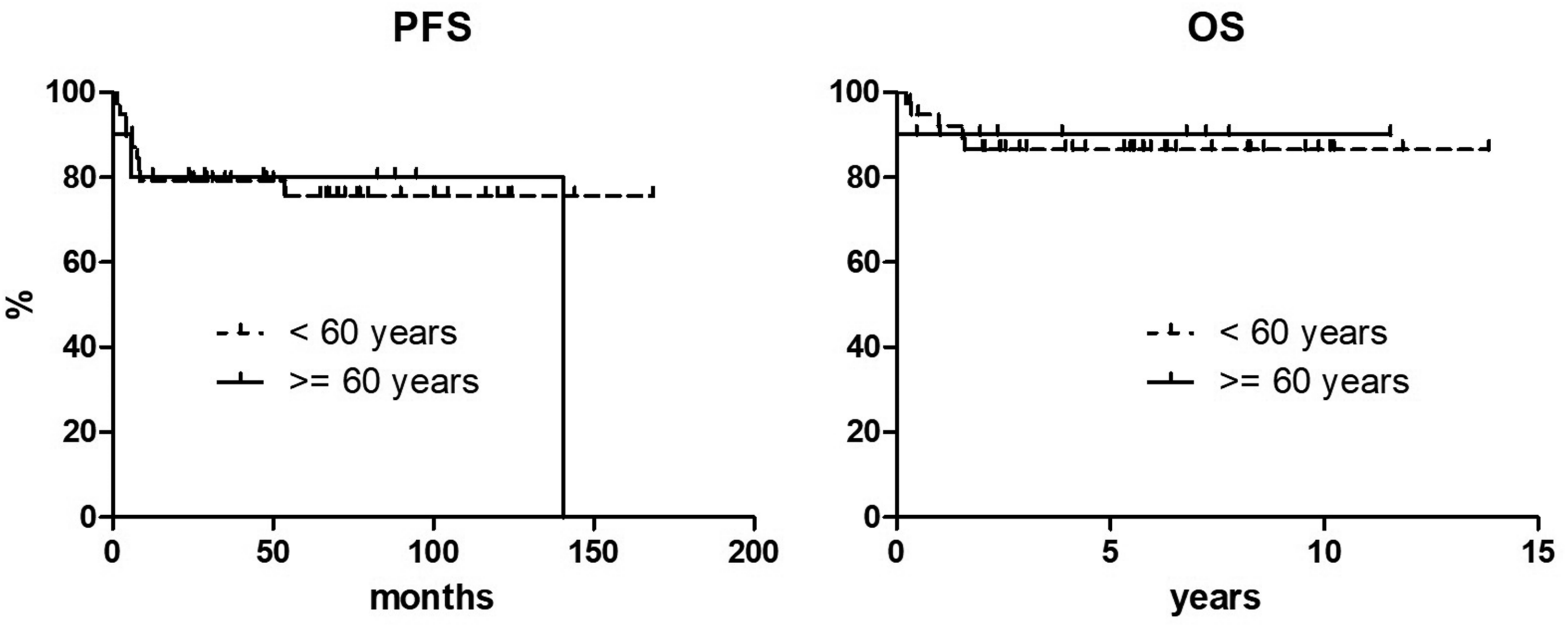
Progression-free and overall survival curves according to age.

**Figure 3.**
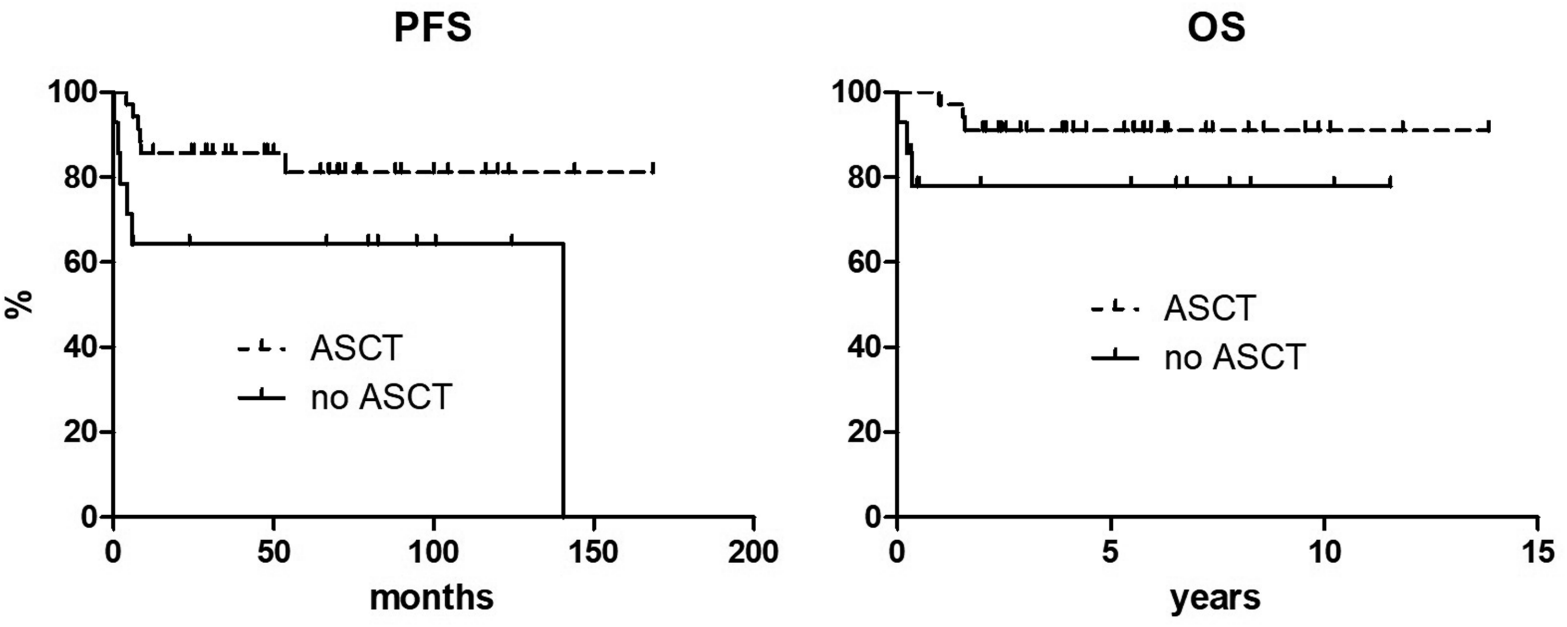
Progression-free and overall survival curves according to transplant status.

No differences in PFS and OS were seen when patients were stratified according to age, assuming 60 years as a cutoff. More specifically, 10-year PFS was 75.6% and 80.0% in patients younger than 60 years and aged 60 years or more, respectively (*P* = 0.699), and 10-year OS was 86.5% and 90.0% for the same age categories (*P* = 0.981) (Fig. 2). When patients were stratified according to ASCT status, the 10-year PFS was 81.2% for patients receiving ASCT and 64.3% for those not undergoing ASCT [*P* = 0.038, hazard ratio (HR) 0.24, 95% HR confidence interval (CI) 0.06-0.92], with both curves reaching a plateau. Six progression events were documented in each group, with an incidence of disease progression of 16.7% in patients receiving ASCT and 42.9% in those not receiving ASCT. Noteworthy, among the patients who did not receive ASCT and that showed disease progression, 3 were younger than 60 years and initially intended to receive consolidative transplant. Similarly, the 10-year OS was 91.1% and 77.9% for the 2 groups, respectively, although the difference was not statistically significant (*P* = 0.078, HR 0.22, 95% HR CI 0.03-1.46) (Fig. 3).

**Figure 4.**
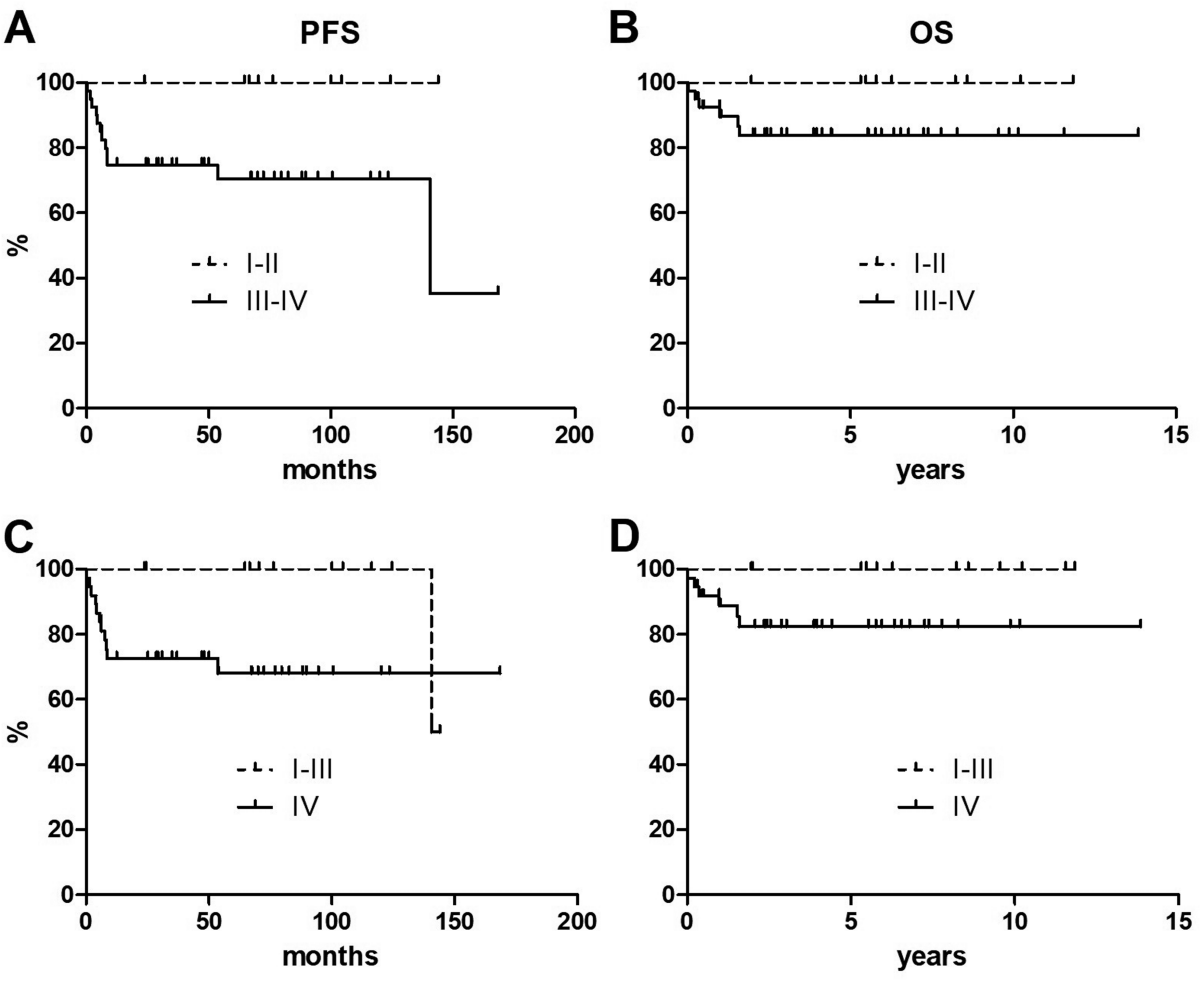
Progression-free (**A-C**) and overall survival (**B-D**) curves according to disease stage at presentation.

Disease stage had an impact in the prognostic stratification of patients. When patients were dichotomized into early- and advanced-stage at presentation (ie, stages I-II versus stages III-IV), the 10-year PFS was 100% versus 70.5%, respectively (*P* = 0.085), and the 10-year OS was 100% versus 83.8%, respectively (*P* = 0.214) (Fig. 4A and 4B). When patients presenting with early-stage and stage III were grouped together and compared to patients with stage IV disease, the difference in terms of PFS at 10 years reached a statistical significance (100% versus 68.1% for each group, respectively, *P* = 0.042) (Fig. 4C). According to the same stratification, the 10-year OS was 100% versus 82.4% for each group, respectively (*P* = 0.134; Fig. 4D).

### Safety

Hematologic AEs represented the most frequent toxicities: grades 3-4 neutropenia, thrombocytopenia, and anemia were seen in 96%, 60%, and 32% of patients, respectively. Infections occurred in 60% of patients, which turned into grade 4 or fatal sepsis in 14% and 8% of cases, respectively. In 10% of the patients, a diagnosis of probable or proven fungal infection was made. Oral mucositis was the most frequent extra-hematologic adverse event, occurring in half of the patients; in 24% of the cases oral mucositis was graded as 3-4. Liver function test alteration was seen in 16% of the patients, with complete recovery upon treatment conclusion. Renal toxicity, in terms of increase of serum creatinine levels, occurred in 6 patients, requiring methotrexate continuous infusion halt in two-thirds of the cases.

Two patients developed a secondary hematologic neoplasm, namely an acute myeloid leukemia (AML) 6 years after ASCT, and a myelodysplastic syndrome 1 year after ASCT. The patient who developed AML could receive adequate induction and consolidation treatment and underwent allogeneic transplantation, obtaining a CR. The patient affected by myelodysplasia was addressed to another center and then was lost to follow up.

## Discussion

Dose-intense multi-agent chemotherapy has always represented the mainstay of treatment of adult BL, a rare but exceptionally aggressive lymphoproliferative disorder. Currently, applied protocols proposed by several international cooperative groups have demonstrated overlapping efficacy and toxicity in real-life settings.^9,11,16-18^ Outcomes are excellent in patients receiving intensive chemoimmunotherapy as confirmed by the low risk of relapse and the life expectancy similar to that of general population in those who achieve a 12-month post-remission event-free survival.^19^ Importantly, high-risk features at diagnosis, identify patients with significantly divergent survival rates.^20,21^ It is hard to define whether ASCT performed as a consolidative strategy in first remission plays a role in improving outcomes, as the experience is limited: a recent meta-analysis underscores the efficacy of ASCT in case of chemosensitive disease, although it remains inconclusive regarding its role as consolidation,^22^ especially in case high-dose regimens are used. Noteworthy, the indication to frontline ASCT has declined significantly over time, with less than 1 out of 5 patients being transplanted later than 2001.^23^

We have presented our 15-year experience with 50 adult BL lymphoma patients treated according to the BFM protocol, with the addition of rituximab. Our data support the feasibility of an age-adapted frontline approach to BL, consisting of a reduced-intensity regimen in elderly people and with the inclusion of ASCT in younger patients. More specifically, 80.3% of the patients are disease-free at 10 years, which indicates a high curability rate of this disease despite the high proportion of advanced stage patients (80.0% among those who were younger than 60 years and 90.0% among elderly patients) and an extranodal disease dissemination in more than 80.0% of the cases. These results compare favorably with previously published experience in patients with BL bearing baseline characteristics that overlap with those described in our series, both in terms of age at presentation and disease burden (advanced stage, number and type of extranodal involvement). Of note, we have detected CNS involvement in only 4.0% of the patients, which is somewhat lower than reported elsewhere.^9,11,16^

We have found no differences in long-term outcomes as far as age is concerned, as more of three-quarters of patients remain progression free at 10 years and up to 90% are alive at the same time point irrespective of being younger or elder than 60 years (Fig. 2). Importantly, no significant increase in AEs or in treatment discontinuation was seen in any of the age categories. This indicates that an age-adapted strategy, based on dose-reduced methotrexate and alkylators and excluding the use of high-dose cytarabine, but with preserved anthracycline dosages, retains its efficacy in patients with predictably reduced BM functionality, without any toxicity excess.

Of note, no PFS or OS events occurred within 10 years since treatment inception in patients with Ann Arbor stages I-II, thus confirming they have reached a cure. Importantly, PFS and OS at 10 years were both 100% even if early-stage was lumped together with stage III, thus underscoring the concept that stage III patients have the same long-term prognosis of early-stage cases when treated with the proposed approach. On the contrary, stage IV patients still display a higher risk of treatment failure, as a consequence of a more disseminated disease, frequently with organ infiltration that hampers their function and impacts on performance status. Disease progression mainly occurs within a few months from treatment initiation due to the aggressiveness of the disease (Fig. 4A and 4C). Nevertheless, the plateau shapes of stage IV PFS curves confirm that a chance for cure is preserved despite early treatment failure in some patients with advanced stage disease. Likewise, the risk of death is maximal in the first 12-24 months since diagnosis, clearly indicating that no rescue strategies appear effective as soon the disease progresses (Fig. 4B and 4D).

A significant difference was observed in PFS between transplanted and non-transplanted patients; the same difference was confirmed in OS, although with no statistical significance. However, both PFS and OS curves display a plateau and indicate that even patients not receiving ASCT may achieve a cure in the long run. The reason of the difference rests on the fact that a proportion of patients who did not receive ASCT displayed a very early disease progression and an early death in most of the cases. In other words, despite being planned to receive ASCT on an intention-to-treat basis, they have never undergone this procedure due to an extremely early treatment failure (Fig. 3). According to this observation, no definite conclusions can be drawn on the need of ASCT as response consolidation, although it may retain a role in patients with some high-risk features at presentation, like diffuse disease spreading or involvement of high-risk sites.

Grades 3-4 hematologic toxic effects, along with mucositis and infections, represent the major concern with the use of high-dose intensity regimens. In order to reduce toxicity, a lower-intensity treatment consisting of continuously infused etoposide, doxorubicin, and vincristine, along with cyclophosphamide, prednisone and rituximab (EPOCH-R) has been proposed.^24^ EPOCH-R was highly effective in BL patients, with both PFS and OS survival rates higher than 95% at a median follow-up of approximately 85 months. Toxic effects were all mild and mainly confined to grades 1 and 2. Of note, patients in trial did not receive any intravenous CNS-directed treatment apart from ITP, as agents able to cross the blood-brain barrier were not part of the infusion protocol.^24^ In a following study of risk-adapted treatment with dose-adjusted (DA)-EPOCH-R, the 4-year event-free survival and OS were 84.5% and 87.0%, respectively, although with much more disappointing results in patients with positive cerebrospinal fluid at baseline (10% of the cases), whose 4-year EFS was 45.5%.^25^ More recently, in a head-to-head comparison of DA-EPOCH-R (6 cycles) and rituximab, cyclophosphamide, doxorubicin, vincristine, methotrexate alternating with rituximab, ifosfamide, etoposide and cytarabine (R-CODOX-M/R-IVAC, 2 cycles), the CR rates and the 2-year PFS and OS rates in both arms were comparable. Importantly, CNS involvement was an exclusion criterion. DA-EPOCH-R however was associated with less infectious complications, transfusion, and days of hospitalization than R-CODOX-M/R-IVAC, thus benchmarking against high-dose intensity regimens, provided CNS is not affected.^26^

## Conclusion

In conclusion, our experience shows that an intensive treatment with the BFM protocol, with rituximab and ASCT, appears feasible, safe and highly effective in adult patients with BL, as confirmed by long-term survival rates. A dose-adapted strategy according to age is active in elderly patients, with a significant amount of them reaching a final cure. ASCT does not seem to enhance patients’ performance in the very long term, and its use should be limited to selected cases, if not omitted at all.

## Supplementary Material

oyae017_suppl_Supplementary_Tables_S1

oyae017_suppl_Supplementary_Figures_S1-S2

## Data Availability

De-identified patient clinicopathologic and therapy-related toxicity data are provided in the manuscript.
