# Supplementary material for: The Treatment of Burkitt Lymphoma With the Berlin-Frankfurt-Münster Protocol With Rituximab and Consolidative Autologous Transplantation
Source: Oncologist. 2024 Feb 10;29(6):e789–95. doi: 10.1093/oncolo/oyae017 (PMC11144971; doi:10.1093/oncolo/oyae017)
Supplement: oyae017_suppl_Supplementary_Tables_S1 [file oyae017_suppl_supplementary_tables_s1.docx]

**Supplementary Table (Methods)**

**Table S1.** Dose details according to age-groups. Dose modifications due to age are highlighted in bold.

|  | **Age < 60** | **Age ≥ 60** |
| --- | --- | --- |
| **Block A**  Rituximab (day 1)  Vincristine (day 2)  Dexamethasone (days 2 → 6)  Ifosfamide (days 2 → 6)  Methotrexate (day 2)  Etoposide (days 5, 6)  Cytarabine (days 5, 6) | 375 mg/m^2^  **2 mg**  10 mg/m^2^  **800 mg/m^2^**  **1,500 mg/m^2^**  **100 mg/m^2^**  **300 mg/m^2^** | 375 mg/m^2^  **1 mg**  10 mg/m^2^  **400 mg/m^2^**  **550 mg/m^2^**  **60 mg/m^2^**  **120 mg/m^2^** |
| **Block B**  Rituximab (day 1)  Vincristine (day 2)  Dexamethasone (days 2 → 6)  Cyclophosphamide (days 2 → 6)  Methotrexate (day 2)  Doxorubicin (days 5, 6) | 375 mg/m^2^  **2 mg**  10 mg/m^2^  200 mg/m^2^  **1,500 mg/m^2^**  25 mg/m^2^ | 375 mg/m^2^  **1 mg**  10 mg/m^2^  200 mg/m^2^  **500 mg/m^2^**  25 mg/m^2^ |
| **Block C**  Rituximab (day 1)  Vindesine (day 2)  Dexamethasone (days 2 → 6)  Methotrexate (day 2)  Etoposide (day 5, 6)  Cytarabine (day 6) | 375 mg/m^2^  3 mg/m^2^  10 mg/m^2^  1,500 mg/m^2^  250 mg/m^2^  4,000 mg/m^2^ | Not given  Not given  Not given  Not given  Not given  Not given |
