# Supplementary figures and images for: The Treatment of Burkitt Lymphoma With the Berlin-Frankfurt-Münster Protocol With Rituximab and Consolidative Autologous Transplantation
Source: Oncologist. 2024 Feb 10;29(6):e789–95. doi: 10.1093/oncolo/oyae017 (PMC11144971; doi:10.1093/oncolo/oyae017)

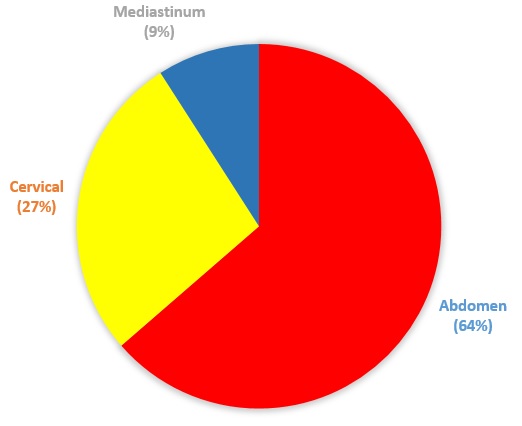

Supplement: oyae017_suppl_Supplementary_Figures_S1-S2 [file oyae017_suppl_supplementary_figures_s1-s2.zip › New folder/Supplementary Figure S1.jpg]

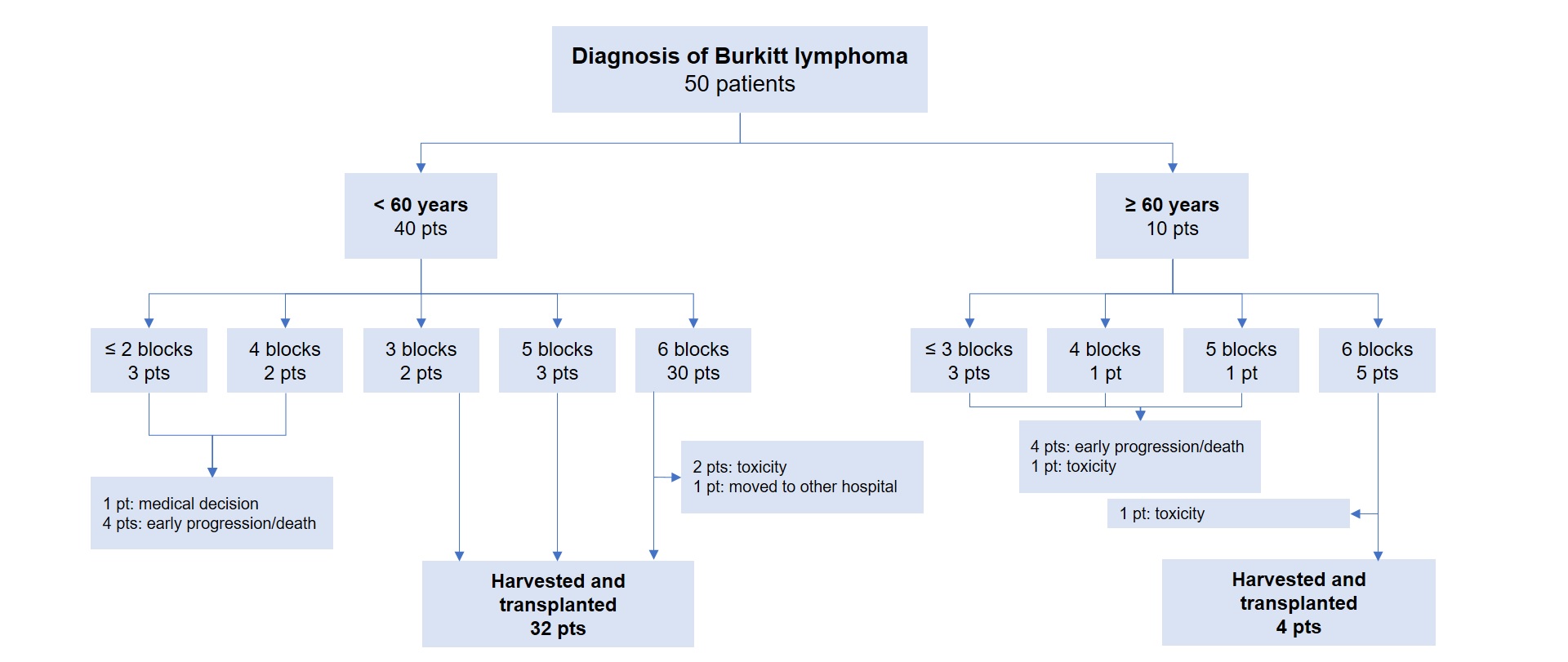

Supplement: oyae017_suppl_Supplementary_Figures_S1-S2 [file oyae017_suppl_supplementary_figures_s1-s2.zip › New folder/Supplementary Figure S2.jpg]
